# Anti-inflammatory compounds reduce equine herpesvirus type 1 replication and cell-to-cell spread

**DOI:** 10.3389/fvets.2023.1165917

**Published:** 2023-05-19

**Authors:** Jeanette B. Black, Arthur R. Frampton

**Affiliations:** Department of Biology and Marine Biology, University of North Carolina Wilmington, Wilmington, NC, United States

**Keywords:** EHV-1, inflammation, anti-inflammatories, epithelial, endothelial

## Abstract

Equine herpesvirus type 1 (EHV-1) is a highly transmissible pathogen that leads to a variety of clinical disease outcomes in infected horses. A major sequela that can occur after an EHV-1 infection is a neurological disease termed equine herpesvirus myeloencephalopathy (EHM). Clinical manifestations of EHM include fever, ataxia, incontinence, and partial to full paralysis, which may ultimately lead to the euthanization of the infected horse. To develop an effective treatment strategy for EHM, it is critical that the specific virus–host interactions that lead to EHM be investigated so that safe and effective therapeutic interventions can be developed and delivered. In this study, we examined the ability of four non-steroidal anti-inflammatory drugs (NSAIDs), a steroidal anti-inflammatory drug (dexamethasone), a Rho-kinase (ROCK) inhibitor, and a JAK/STAT inhibitor (AG490) to reduce EHV-1 virus yields and cell-to-cell spread. We show that the NSAID, flunixin meglumine (FM), and the JAK/STAT inhibitor, AG490, significantly reduced virus yields in endothelial and epithelial cell lines, and this inhibition was similar for two neurologic and two non-neurologic EHV-1 strains. In addition to reducing virus yields, AG490 and FM also significantly reduced the ability of EHV-1 to spread laterally from cell to cell.

## Introduction

Equine herpesvirus type 1 (EHV-1) is a ubiquitous virus that causes considerable morbidity and mortality in horses (1–3). Most horses are infected with EHV-1 in their first year of life and exhibit clinical signs that include respiratory distress, mucopurulent discharge, fever, and malaise. In addition to infection in the upper respiratory tract, EHV-1 can also cause abortigenic and/or neurologic disease. Disease outcomes or sequelae associated with EHV-1 can vary quite dramatically depending on various host and viral factors. One viral determinant that is positively correlated with a neurologic disease outcome is the presence of a point mutation within the viral DNA polymerase gene of the infecting strain (4–7). EHV-1 strains that contain a point mutation (A2254 > G2254) within the DNA polymerase (DNA Pol) gene have been classified as neurologic, while those that do not harbor this mutation are classified as non-neurologic. Data collected from various groups have shown that EHV-1 strains with this DNA Pol mutation are responsible for the majority of EHM cases (5, 8). EHV-1 strains that harbor this mutation replicate more efficiently in peripheral blood mononuclear cells (PBMCs) and establish a longer viremia, which may result in an increased ability of EHV-1 to reach and productively infect the vascular endothelium in the CNS (9–11).

The increased infection of the CNS endothelium leads to the induction and release of significant amounts of pro-inflammatory cytokines from endothelial cells which results in the recruitment of lymphocytes and natural killer (NK) cells to the infected site. This hyper-inflammation causes thrombosis, ischemia, and hypoxia in the CNS and damages neuronal cells (12–15). The increased incidence of EHM observed in horses infected with a neurologic strain led us to hypothesize that the neurologic strains would induce higher levels of damaging pro-inflammatory cytokines compared to the non-neurologic strains. However, in a previous study (16), when we examined the expression of 49 host immune genes in EHV-1 infected endothelial cells, we observed similar expression of these genes in cells infected with neurologic and non-neurologic strains. Therefore, these results indicate that the induction of pro-inflammatory cytokines is similar from one strain to the next and suggest that any EHV-1 strain, regardless of their designation as neurologic or non-neurologic, can cause inflammation that contributes to EHM when the virus can gain access to the CNS.

Due to the hyper-inflammation associated with EHV-1, various anti-inflammatory drugs have been used as part of a therapeutic regimen to treat EHV-1-infected horses (12). While these drugs were developed to target inflammation, studies have also shown that some anti-inflammatory drugs have the added benefit of blocking or reducing the replication and spread of multiple herpesviruses, such as pseudorabies virus (PRV) (17, 18), Marek's disease virus (MDV) (19), and cytomegalovirus (CMV) (20, 21). In addition to reducing replication and cell-to-cell spread, inhibition of the inflammatory mediator cyclooxygenase-2 (COX-2) also reduced the lytic reactivation of Epstein–Barr virus (EBV).

JAK/STAT inhibitors have also been shown to inhibit a variety of viruses. HIV replication (22), viral reservoir establishment (23), and encephalitis (24) have all been reduced by JAK/STAT inhibitors. In addition to HIV, Zika, and SARS-CoV-2, virus replication was also inhibited in the presence of JAK/STAT inhibitors (25, 26). Previous studies have also shown that the level of EHV-1 infection is negatively impacted by the host interferon response and that exogenous administration of recombinant equine interferon alpha reduced EHV-1 replication in primary equine respiratory epithelial cells (27). Together, these drug studies provide evidence that anti-inflammatory drugs, in addition to reducing inflammation, also have the added benefit of interfering with host cell factors that contribute to optimum virus infectivity, replication, and propagation. Based on data collected from these drug studies, we hypothesized that the administration of anti-inflammatory drugs and JAK/STAT inhibitors would reduce EHV-1 viral replication and cell-to-cell spread. To test this hypothesis, we used a cell culture model to investigate the ability of multiple compounds to reduce EHV-1 replication and cell-to-cell spread. We show that both the JAK/STAT pathway inhibitor, AG490, and the NSAID flunixin meglumine (FM) significantly inhibited virus replication and reduced cell-to-cell spread.

## Materials and methods

### Cells, viruses, and compounds

Rabbit kidney-13 (RK-13) cells were provided by Dennis O'Callaghan (Louisiana State University Health Sciences Center, Shreveport, Louisiana, USA), and equine cardiac artery endothelial (EE) cells were provided by Udeni Balasuriya (Louisiana State University, Baton Rouge, Lousiana, USA). All cells were grown in Dulbecco's Modified Eagle's Medium (DMEM) supplemented with 10% fetal bovine serum (FBS) and 2% of 10,000 U/ml penicillin and 10,000 μg/ml streptomycin in normal saline. Cells were cultured at 37°C in 5% CO_2_. EHV-1 strains T220, KyA, T953, and T967 were used in this study. T220 and KyA are non-neurologic, and T953 and T967 are neurologic strains based on the absence or presence, respectively, of the DNA polymerase mutation (A2254 > G2254). T220 and T967 were provided by Udeni Balasuriya. KyA was provided by Dennis O'Callaghan, and T953 (Ohio 2003) was provided by Gillian Perkins (Cornell University, Ithaca, New York, USA). Compounds tested include AG490 (JAK/STAT inhibitor), flunixin meglumine, ketoprofen, phenylbutazone, firocoxib (NSAIDs), and Y-27632 (Rho-kinase inhibitor). All compounds were purchased from Avantor (Avantor, Radnor, Pennsylvania, USA).

### Virus yield assays

EE and RK-13 cells were seeded at 2.5 × 10^5^ cells/well in a 24-well plate. At 24 h post-seeding, cells were treated with 100 μM each of AG490, flunixin meglumine, dexamethasone, ketoprofen, phenylbutazone, Y-27632, and firocoxib in DMEM containing 5% FBS. Control cells were treated with 1% DMSO. Cells were incubated for 24 h at 37°C and 5% CO_2_. Twenty-four hours after drug treatment, cells were infected with each EHV-1 strain at an MOI of 0.1 or 1 and incubated at 37°C and 5% CO_2_ for 24 h. Twenty-four hours post-infection, the supernatants were collected, and then 100 μl of trypsin was added to each well to detach any cells adhering to the well. The trypsin containing the cells was then added to the supernatants, and the samples were centrifuged at 13,000 RPM for 10 minutes. Supernatants were transferred to new 1.7-ml microcentrifuge tubes and stored on ice. The remaining cell pellets were re-suspended in 100 μl of their respective supernatant, and the samples were subjected to three freeze–thaw cycles between −80 and 37°C to release cell-associated virus. After three freeze–thaw cycles, the disrupted cell pellets were added back to their respective supernatants, and the samples were stored at −80°C.

Each sample was thawed in a 37°C water bath, and then 10-fold dilutions of each sample were made in DMEM. In total, 100 μl of each dilution was added to confluent monolayers of RK-13 cells in a 24-well plate, after which the titer plates were incubated at 37°C and 5% CO_2_. After a 1.5-h attachment period, cells were overlaid with 1 ml of semi-solid DMEM containing 1% methylcellulose and 10% FBS, and the cells were incubated for 72 h at 37°C and 5% CO_2_. After the 72 h incubation, the semi-solid medium was removed by aspiration, and the cells were stained with 1% crystal violet for 1 h. Crystal violet was rinsed off the cells, and the plates were inverted and allowed to dry overnight. The following day, plaques were counted, and the titers were determined for each sample and recorded as plaque-forming units per ml (pfu/ml). Three biological replicates were run for each condition. The mean titers of the three biological replicates of the drug-treated cells were compared to the non-drug-treated (1% DMSO) cells, and a Student's *t*-test was performed to determine whether the mean virus titers were significantly different between the two groups. *p-*values of ≤ 0.05 were considered statistically significant.

AG490 and FM were also added to RK-13 cells post-infection. RK-13 cells were seeded at 2.5 × 10^5^ cells/well in a 24-well plate and then infected with each EHV-1 strain at an MOI of 0.1 for 24 h at 37°C and 5% CO_2_. Twenty-four hours post-infection, supernatants, and cell-associated viruses were collected and combined as described for the pre-drug treatment assays. Three biological replicates were run for each condition, and the mean titers of the three biological replicates of the drug-treated cells were compared to the non-drug-treated (1% DMSO) cells. A Student's *t*-test was performed to determine whether the mean virus titers were significantly different between the two groups. *p-*values of ≤ 0.05 were considered statistically significant.

### Cell-to-cell spread assays

RK-13 cells were seeded at 5 × 10^5^ cells/well in 6-well plates which were then infected the next day with 100 pfu of T967, T220, T953, or KyA for 2 h. Following infection, media and virus were aspirated from each well, and a 1% methylcellulose solution containing 10% FBS was overlaid on the cell monolayers. Infected cells were incubated for 24 h at 37°C and 5% CO_2_, then each compound (100 μM) was mixed with the methylcellulose solution, added to the cells, and the cells were incubated for 24 h at 37°C and 5% CO_2._ After 24 h, another dose (100 μM) of the compound was added, and the cells were incubated for another 24 h at 37°C and 5% CO_2._ At 72 h post-infection, methylcellulose was aspirated from the wells, and the cells were fixed and stained with 1% crystal violet for 1 h and then rinsed with water. The wells were allowed to dry overnight, and the plaques were imaged using an Olympus Fluoview microscope. For each drug-treated group, a total of eight images (four quadrants per image) were processed using the Java-based image processing program, Image J (IJ), and ViralPlaque software. The size of the plaques in the drug-treated, infected cells was analyzed and compared with the size of the plaques that formed in the 1% DMSO control group. For each treatment, a total of 100 plaques were measured to determine the mean, standard deviation, and standard error.

## Results

### Drug inhibition of the virus yields pre-infection

Five anti-inflammatory drugs (flunixin meglumine, dexamethasone, firocoxib, ketoprofen, and phenylbutazone), a Rho-kinase (ROCK) inhibitor (Y-27632), and a JAK/STAT inhibitor (AG490) were investigated for their ability to reduce EHV-1 replication and cell-to-cell spread. These drugs were tested against four EHV-1 strains, on two cell lines (epithelial and endothelial), at two multiplicities of infection (MOIs). The EHV-1 strains consisted of two non-neurological strains, T220 and KyA, and two neurological strains, T953 (Ohio 2003) and T967. In the initial virus yield assay, equine endothelial (EE) and rabbit kidney (RK-13) epithelial cells were treated with each drug for 24 h and then infected with each EHV-1 strain at a multiplicity of infection (MOI) of 0.1 and 1 for 24 h; then, the total amount of virus produced (virus yield) was measured as described in the Materials and methods section. All experiments were run in triplicate (three biological runs), and the mean virus yields in each group were compared to the control cells that were only given the solvent DMSO and infected with EHV-1.

**Figure 1:**
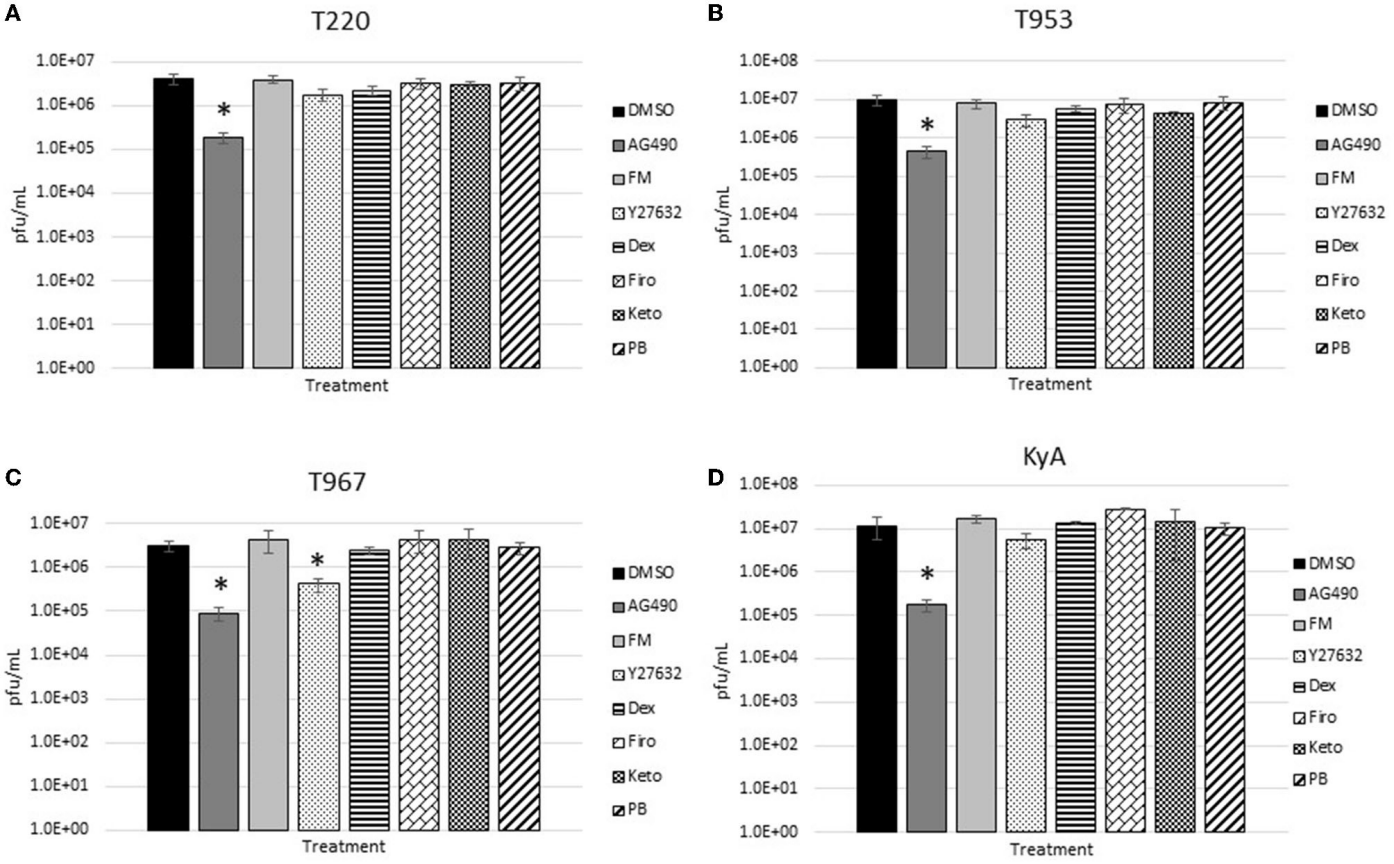
Equine endothelial (EE) cells (5 × 10^5^ cells/well) were treated with drugs (100 μM) for 24 h and then infected with EHV-1 strains **(A)** T220, **(B)** T953, **(C)** T967, and **(D)** KyA at an MOI of 0.1 for 24 h in the presence of freshly added drug (100 μM). After 24 h from infection, the total virus was harvested and titered on rabbit kidney-13 (RK-13) cells. Three independent assays were run for each strain and drug, and the mean titers were calculated from the three biological assays. Virus yields in the drug-treated cells were compared to cells that were treated with the solvent, 1% DMSO, and infected with EHV-1. A Student's *t*-test was performed, and *p*-values of ≤0.05 were considered to be significant. *p-values ≤ 0.05.

**Figure 2:**
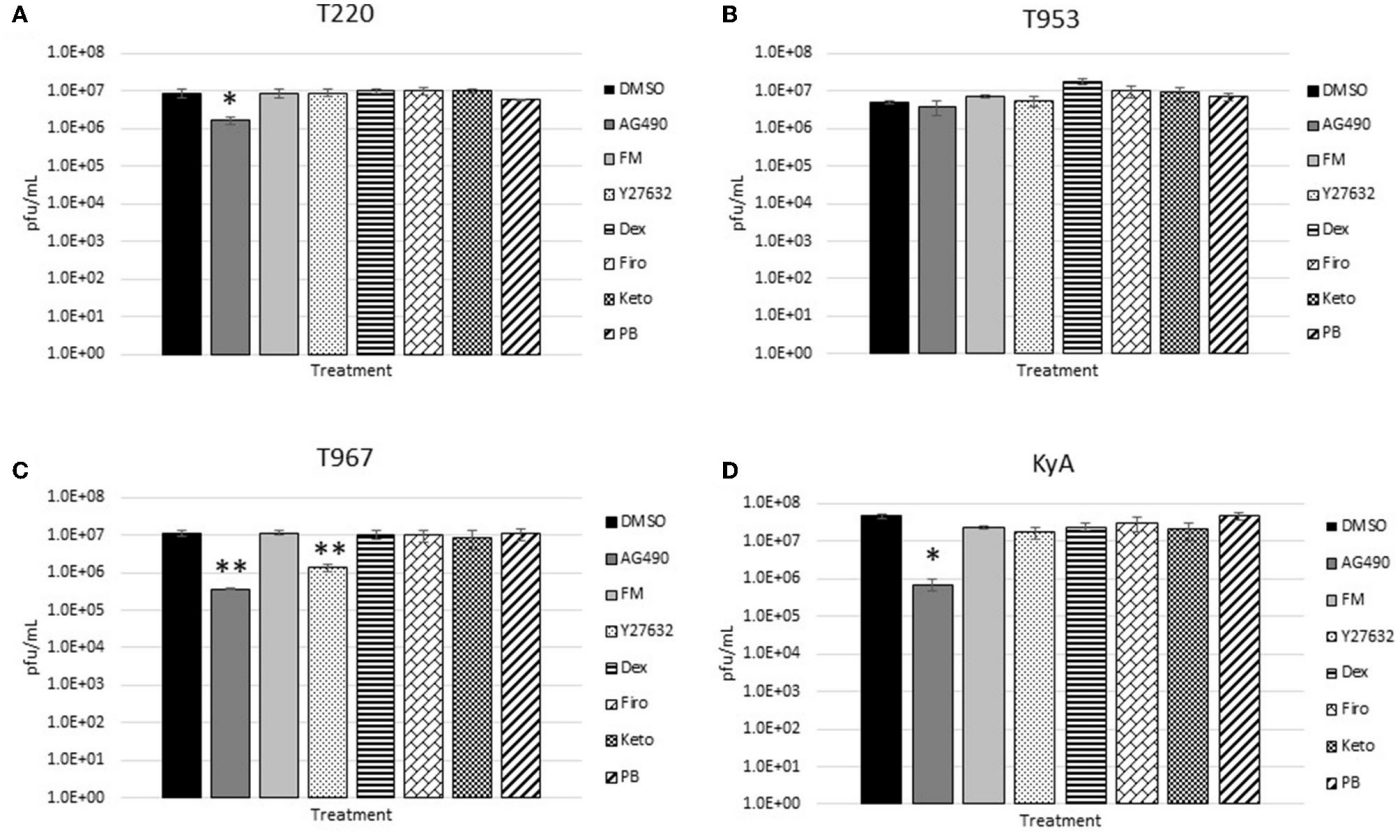
Equine endothelial (EE) cells (5 × 10^5^ cells/well) were treated with drugs (100 μM) for 24 h and then infected with EHV-1 strains **(A)** T220, **(B)** T953, **(C)** T967, and **(D)** KyA at an MOI of 1 for 24 h in the presence of freshly added drug (100 μM). After 24 h from infection, the total virus was harvested and titered on rabbit kidney-13 (RK-13) cells. Three independent assays were run for each strain and drug, and the mean titers were calculated from the three biological assays. Virus yields in the drug-treated cells were compared to cells that were treated with the solvent, 1% DMSO, and infected with EHV-1. A Student's *t*-test was performed, and *p*-values of ≤0.05 were considered to be significant. *p-values ≤ 0.05 and **p-values ≤ 0.01.

**Figure 3:**
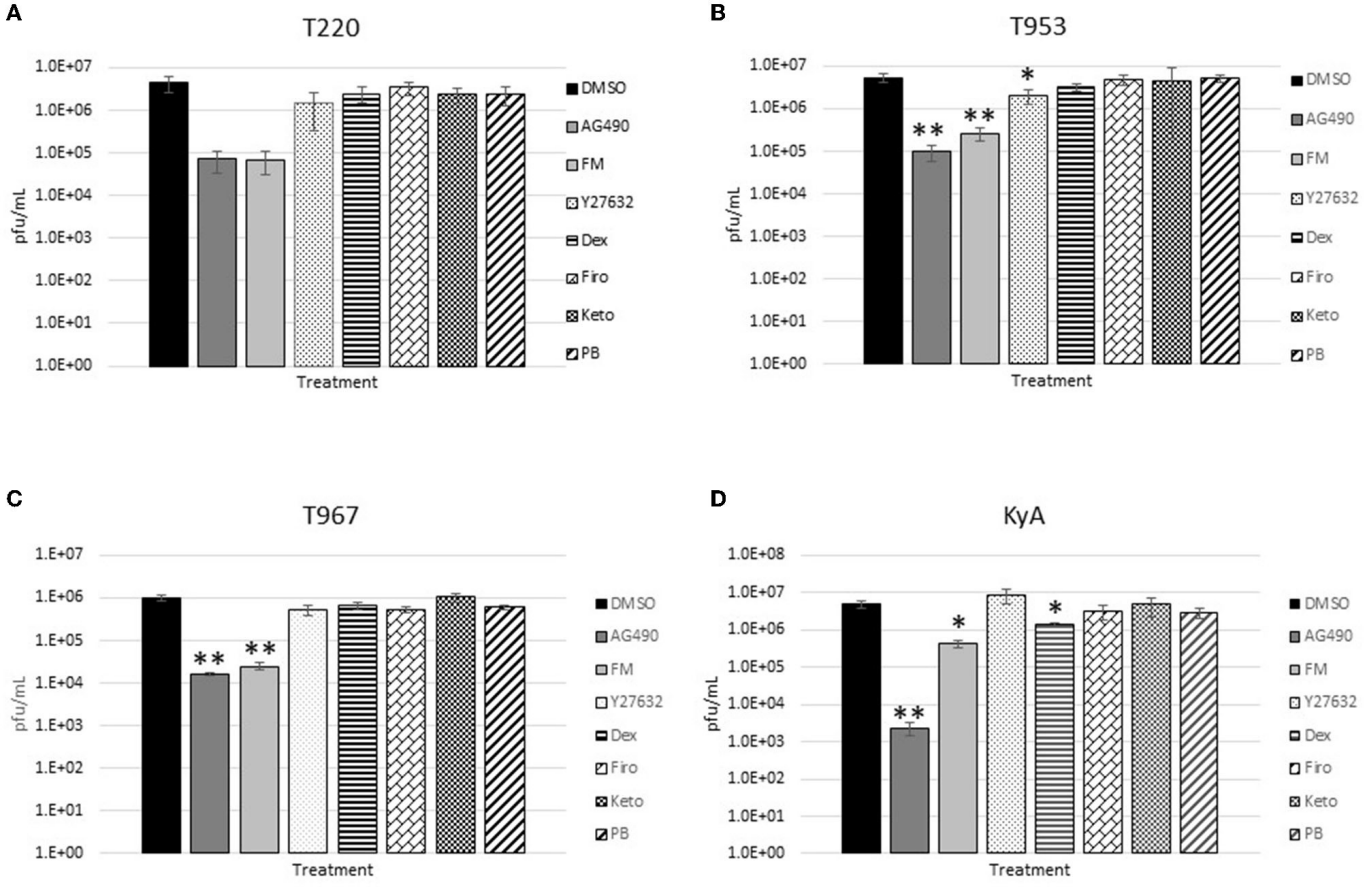
Equine endothelial (EE) cells (5 × 10^5^ cells/well) were treated with drugs (100 μM) for 24 h and then infected with EHV-1 strains **(A)** T220, **(B)** T953, **(C)** T967, and **(D)** KyA at an MOI of 0.1 for 24 h in the presence of freshly added drug (100 μM). After 24 h from infection, the total virus was harvested and titered on rabbit kidney-13 (RK-13) cells. Three independent assays were run for each strain and drug, and the mean titers were calculated from the three biological assays. Virus yields in the drug-treated cells were compared to cells that were treated with the solvent, 1% DMSO, and infected with EHV-1. A Student's *t*-test was performed, and *p*-values of ≤0.05 were considered to be significant. *p-values ≤ 0.05 and **p-values ≤ 0.01.

**Figure 4:**
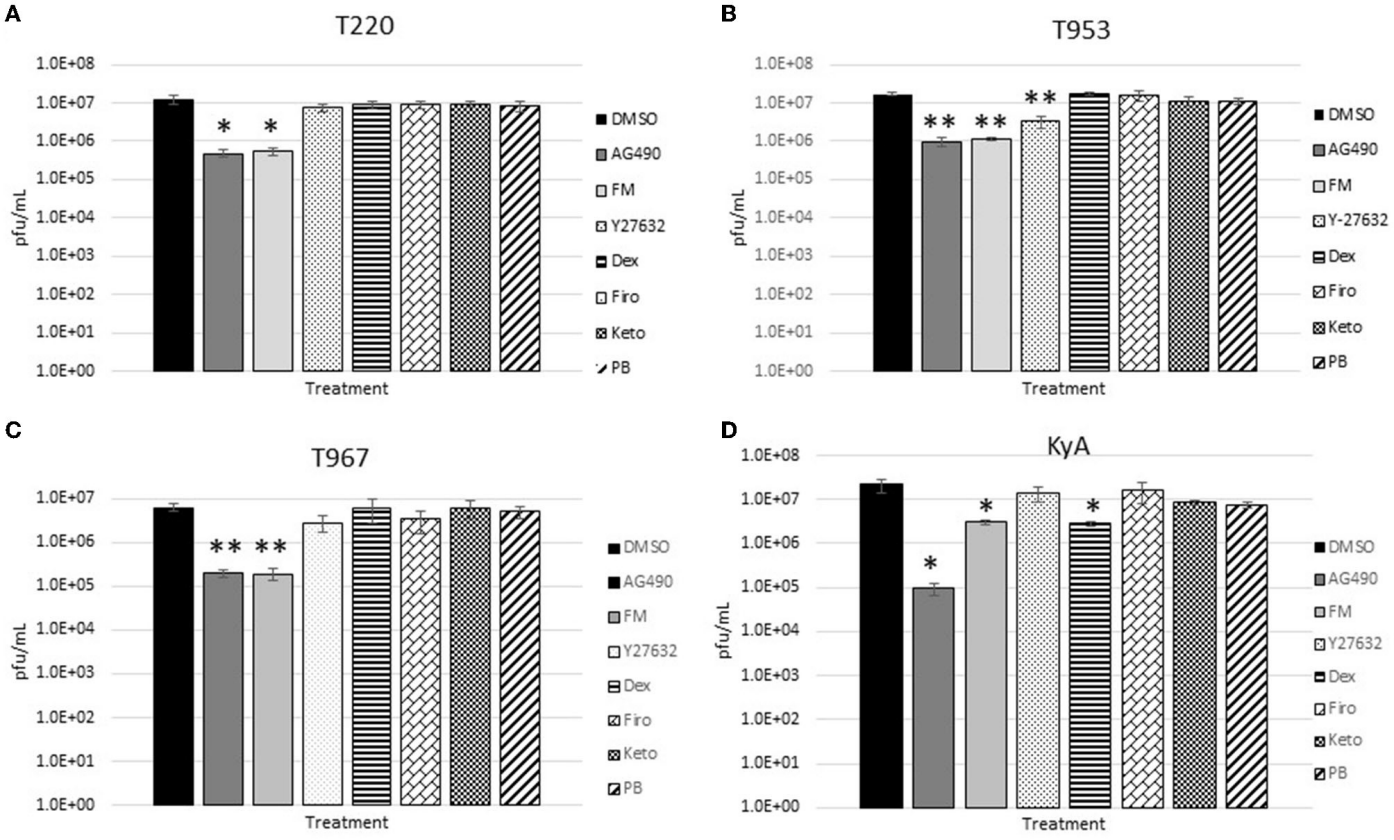
Rabbit kidney-13 (RK-13) epithelial cells (5 × 10^5^ cells/well) were treated with drugs (100 μM) for 24 h and then infected with EHV-1 strains **(A)** T220, **(B)** T953, **(C)** T967, and **(D)** KyA at an MOI of 1 for 24 h in the presence of freshly added drug (100 μM). After 24 h from infection, the total virus was harvested and titered on rabbit kidney-13 (RK-13) cells. Three independent assays were run for each strain and drug, and the mean titers were calculated from the three biological assays. Virus yields in the drug-treated cells were compared to cells that were treated with the solvent, 1% DMSO, and infected with EHV-1. A Student's *t*-test was performed, and *p*-values of ≤0.05 were considered to be significant. *p-values ≤ 0.05 and **p-values ≤ 0.01.

The measured virus yields are shown in Figures 1–**4**. Figure 1 shows the 24 h yields obtained from EE cells that were infected with an MOI of 0.1, and Figure 2 shows the 24 h yields obtained from EE cells infected at an MOI of 1. Figure 3 shows the 24 h yields obtained from RK-13 cells that were infected at an MOI of 0.1, and Figure 4 shows the 24 h yields obtained from RK-13 cells infected at an MOI of 1.

Results obtained from the virus yield assays showed that the JAK/STAT inhibitor, AG490, was the only drug that demonstrated significant antiviral activity against all EHV-1 strains in equine endothelial (EE) cells. In EE cells infected at an MOI of 0.1 (Figure 1), AG490 reduced T220 virus yields by 23-fold, T953 virus yields by 22-fold, T967 virus yields by 35-fold, and KyA virus yields by 68-fold. At an MOI of 1 (Figure 2), AG490 reduced T220 yields by 5-fold, T967 yields by 31-fold, and KyA yields by 67-fold. Interestingly, we observed no difference in virus yields in AG490-treated EE cells infected with T953 at an MOI of 1. The Rho-kinase inhibitor, Y-27632, also exhibited significant antiviral activity against T967 at both MOIs.

In the RK-13 epithelial cell line, antiviral activity was also observed in cells treated with AG490. In these cells, the overall inhibitory activity was more robust compared to that observed in EE cells. In RK-13 cells infected at an MOI of 0.1 (Figure 3), AG490 reduced T953 virus yields by 53-fold, T967 by 61-fold, and KyA by 212-fold. A reduction was also observed in T220 virus yields at an MOI of 0.1, but the *p*-value was 0.08. In RK-13 cells infected at an MOI of 1 (Figure 4), AG490 reduced T220 virus yields by 25-fold, T953 by 18-fold, T967 by 31-fold, and KyA by 229-fold.

In addition to AG490, we also observed virus inhibition against all EHV-1 strains in RK-13 cells with the NSAID flunixin meglumine (FM). In RK-13 cells infected at an MOI of 0.1 (Figure 3), FM reduced T953 virus yields by 20-fold, T967 by 41-fold, and KyA by 12-fold. Similar to AG490, FM also reduced T220 virus yields at an MOI of 0.1, but the *p*-value was 0.08. In RK-13 cells infected at an MOI of 1 (Figure 4), FM-reduced T220 virus yields by 25-fold, T953 by 15-fold, T967 by 33-fold, and KyA by 7-fold. In addition to AG490 and FM, Y-27632 also significantly reduced T953 virus yields at an MOI of 0.1, and dexamethasone inhibited KyA virus yields at both MOIs.

### Drug inhibition of virus yields after infection

**Figure 5:**
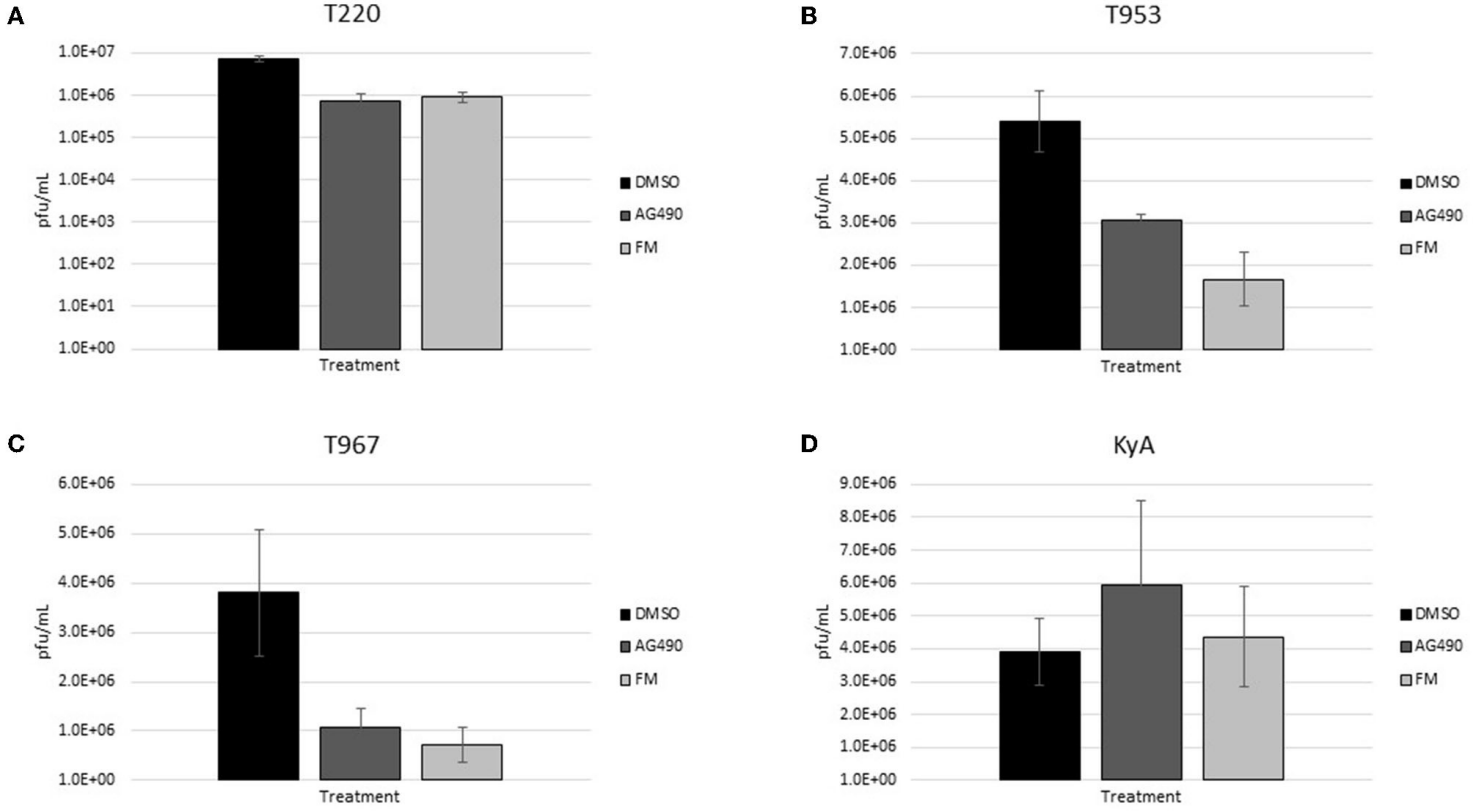
Rabbit kidney-13 (RK-13) epithelial cells (5 × 10^5^ cells/well) were treated with drugs (100 μM) for 24 h and then infected with EHV-1 strains **(A)** T220, **(B)** T953, **(C)** T967, and **(D)** KyA at an MOI of 1 for 24 h in the presence of freshly added drug (100 μM). After 24 h from infection, the total virus was harvested and titered on rabbit kidney-13 (RK-13) cells. Three independent assays were run for each strain and drug, and the mean titers were calculated from the three biological assays. Virus yields in the drug-treated cells were compared to cells that were treated with the solvent, 1% DMSO, and infected with EHV-1. A Student's *t*-test was performed, and *p*-values of ≤0.05 were considered to be significant. *p-values ≤ 0.05 and **p-values ≤ 0.01.

Based on the ability of AG490 and FM to reduce virus yields in RK-13 epithelial cells when administered prior to infection, we investigated whether either of these compounds would be able to reduce EHV-1 yields when added post-infection. For these assays, RK-13 cells were infected with each EHV-1 strain at an MOI of 0.1 for 24 h; then, AG490 and FM were added to the cells for a further 24 h, after which the virus was harvested and titered to determine virus yields. As shown in Figure 5, post-infection administration of both drugs reduced virus yields in T220, T953, and T967-infected cells, while no inhibition was observed in KyA-infected cells. AG490 reduced T220 virus yields by 10-fold, T953 by 1.8-fold, and T967 by 4-fold. FM reduced T220 virus yields by 8-fold, T953 by 3-fold, and T967 by 5-fold.

### Drug inhibition of the cell-to-cell viral spread

**Figure 6:**
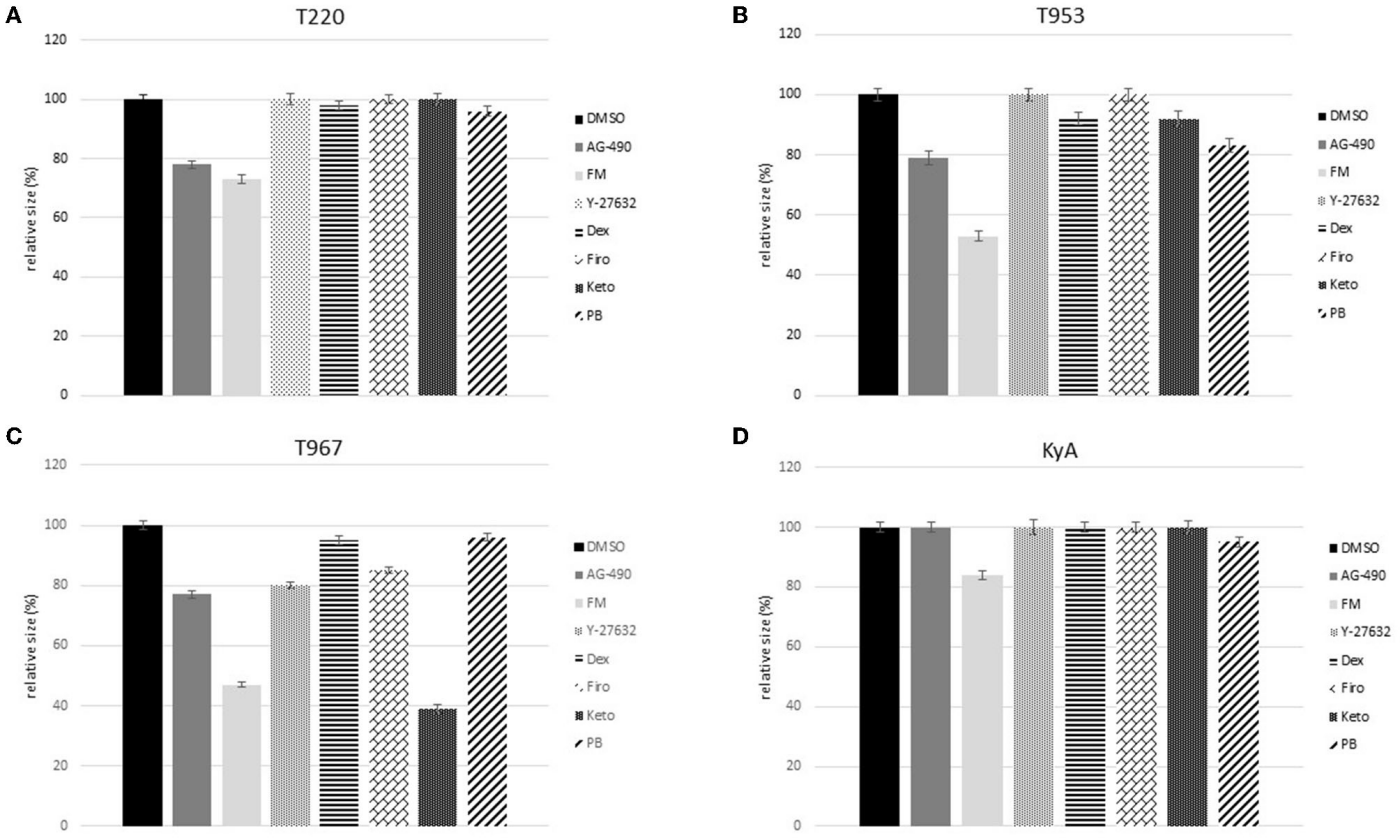
Rabbit kidney-13 (RK-13) epithelial cells (1 × 10^6^ cells/well) were infected with 100 plaque-forming units (pfu) of **(A)** T220 **(B)** T953, **(C)** T967, and **(D)** KyA for 2 h. Following infection, media and virus were aspirated, and a 1% methylcellulose solution was overlaid on the cell monolayers. Infected cells were incubated for 24 h, then each drug (100 μM) was added, and the cells were incubated for a further 24 h, after which another dose (100 μM) of the drug was added, and the cells were incubated for an additional 24 h. At 72 h post-infection, the cells were fixed and stained with 1% crystal violet, and the size of the plaques in the drug-treated, infected cells was analyzed and compared to that of the plaques that formed in the control, 1% DMSO, treated, and infected groups. For each treatment, a total of 100 plaques were measured to determine the mean, standard deviation, and standard error.

In addition to evaluating drugs for their ability to reduce virus production, we also examined the ability of these drugs to reduce the amount of cell-to-cell spread by assessing the size of plaques that form in a cell monolayer (28, 29). Smaller plaques indicate that the virus is less able to spread from cell to cell. To test this hypothesis, we treated cells with the same panel of compounds used for the virus yield assays to test whether any of these compounds could effectively reduce cell-to-cell spread. As shown in Figure 6, AG490 significantly reduced the size of plaques formed by all four virus strains compared to the cells that were not treated with the drug (DMSO). We saw a similar but not as dramatic reduction in virus yields in response to the treatment of cells with flunixin meglumine after infection. A second NSAID, ketoprofen, significantly limited the cell-to-cell spread of one strain, T967. These data indicate that in addition to limiting virus replication and yield, FM, and AG490 also significantly limited the ability of each EHV-1 strain to spread from cell to cell, while the NSAID ketoprofen reduced the spread of strain T967.

## Discussion

Due to the hyper-inflammation observed in horses suffering from equine herpesvirus myeloencephalopathy (EHM), new treatment strategies that can be developed to reduce EHV-1 replication and subsequent inflammation are worth investigating. Multiple anti-inflammatory drugs have been shown to inhibit a wide array of viruses. Drugs that inhibit host cell mediators of inflammation have been reported to block or reduce the replication and/or spread of several herpesviruses, such as pseudorabies virus (PRV) (63, 64), Marek's disease virus (MDV) (65), and cytomegalovirus (66, 67). In addition to reducing replication and cell-to-cell spread, inhibition of the inflammatory mediator cyclooxygenase-2 (COX-2) also decreased the lytic reactivation of Epstein–Barr virus (EBV). JAK/STAT inhibitors have also been shown to exhibit antiviral activity against numerous viruses including HIV (22–24), Zika (25), and SARS-CoV-2 (26).

The ability of anti-inflammatory drugs to reduce inflammation, caused by a variety of stimuli in equines has been widely reported (30, 31). Non-steroidal anti-inflammatory drugs (NSAIDs), including ketoprofen, flunixin meglumine, firocoxib, and phenylbutazone, are commonly prescribed to treat inflammation in horses and have been shown to be very safe (12, 32). These drugs have been evaluated in numerous studies and have been shown to reduce the pathology observed in horses infected with other microbial pathogens including equine protozoal myeloencephalitis (EPM) (33). However, while NSAIDs have been used extensively in equines, they have not been rigorously evaluated for their ability to reduce virus replication and inflammation, which are positively correlated with EHM. Currently, there are very limited data on the drugs that have been administered to horses suffering from EHM. In one study, flunixin meglumine, firocoxib, and dexamethasone were shown to suppress the transfer of EHV-1 from infected peripheral blood monocytes (PBMC) to endothelial cells (34), but no data were collected on the ability of these drugs to reduce virus replication. Other studies have shown that the administration of drugs that enhance the host interferon response limits EHV-1 replication and spread (27, 35, 36). In addition, there have been no studies conducted to evaluate whether pre-exposure prophylactic treatment reduces EHV-1 infection or ameliorates EHV-1-induced pathology.

Equine herpesvirus myeloencephalopathy (EHM) is a disease primarily caused by severe inflammation within the CNS. Infection of endothelial cells as a result of cell-associated viremia results in the production of pro-inflammatory cytokines that leads to immune cell infiltration and localized thrombosis and hypoxia that damages adjacent neurons. The degree of CNS pathology determines the extent of EHM disease and ultimately whether a horse will recover or be euthanized due to paralysis (2, 24, 37, 38). Numerous studies have shown a positive correlation between the degree of viremia and negative disease outcomes following EHV-1 infection (10, 11). Therefore, any therapy that can effectively reduce the level of viremia should also decrease the risk of an EHV-1-infected horse developing EHM.

The data collected in this study show that specific compounds reduced the number of progeny virions produced in epithelial and endothelial cells. Data collected from the virus yield assays show that administration of both the JAK/STAT inhibitor, AG490, and the NSAID, flunixin meglumine (FM), significantly reduced the amount of virus produced in epithelial cells. In addition, AG490 reduced virus yields in both epithelial and endothelial cells while FM reduced virus production in epithelial cells but not in endothelial cells. The Rho-kinase inhibitor, Y-27632, which we have previously shown to inhibit viral movement to the nucleus (39, 40), also exhibited antiviral activity against T967 in endothelial cells and T953 in epithelial cells. The only other drug that reduced viral yields was dexamethasone, which had antiviral activity against KyA at both MOIs in epithelial cells.

To evaluate the ability of these drugs to reduce virus replication post-infection, FM and AG490 were administered 24 h after EHV-1 infection and viral yields were measured and compared to control-treated cells at 24 h post-drug administration. The results of these assays showed that both drugs exhibit antiviral activity even after the cells have been infected. Our data showing that these two drugs limit EHV-1 production both pre- and post-infection make them interesting candidates for further testing in horses, as they may prove to be an additional therapeutic option to consider during an EHV-1 outbreak. The ability of these two drugs, which have different mechanisms of action, to reduce the amount of virus produced in cells would be expected to result in a more positive outcome in an EHV-1-infected horse as the level of virus in the bloodstream (viremia), would be lowered, and previous studies have shown that the level of viremia is directly correlated with disease outcome (10, 11).

As with any model system, there are some limitations to the current study. First, the epithelial cells are a continuous rabbit kidney cell line. While these cells have been extensively used in EHV-1 research, data collected using these cells should be compared to data obtained from equine epithelial cells, ideally, equine cells isolated from the upper respiratory tract (URT). Mucosal explant/primary respiratory epithelial tissue culture systems have been developed (27, 41), and a similar study could be conducted using this system to see whether the reduction in EHV-1 replication and cell-to-cell spread with FM and AG490 is replicated in this system. Second, while a significant amount of data can be collected using continuous cell lines, an investigation of these drugs in the whole animal should be conducted. These studies could be performed either in a mouse model system like the one we previously developed (37) or ultimately in horses.

The results obtained in this study provide evidence that prophylactic and post-exposure administration of FM and AG490 may provide some protection against EHV-1 infection. In terms of practice, one could envision a scenario in which routine surveillance identifies a single or a few horses infected with EHV-1, which would trigger the prophylactic treatment of the remaining horses in the group. In addition, although we did not address this in our current study, other studies have shown that DNA chain-terminating drugs such as valacyclovir (38) and valganciclovir (42) are effective in reducing EHV-1 infection and viremia, and thus, it would be interesting to see if a synergistic reduction of EHV-1 would be observed in horses treated with a combination of a specific antiviral such as ganciclovir and FM or AG490.

## Data availability statement

The raw data supporting the conclusions of this article will be made available by the authors, without undue reservation.

## Author contributions

All authors contributed to developing the research questions, designing the experiments, conducted the research to collect the data, analyzed the data, and wrote the manuscript. All authors contributed to the article and approved the submitted version.
