# Supplementary figures and images for: Anti-inflammatory compounds reduce equine herpesvirus type 1 replication and cell-to-cell spread
Source: Front Vet Sci. 2023 May 19;10:1165917. doi: 10.3389/fvets.2023.1165917 (PMC10235532; doi:10.3389/fvets.2023.1165917)

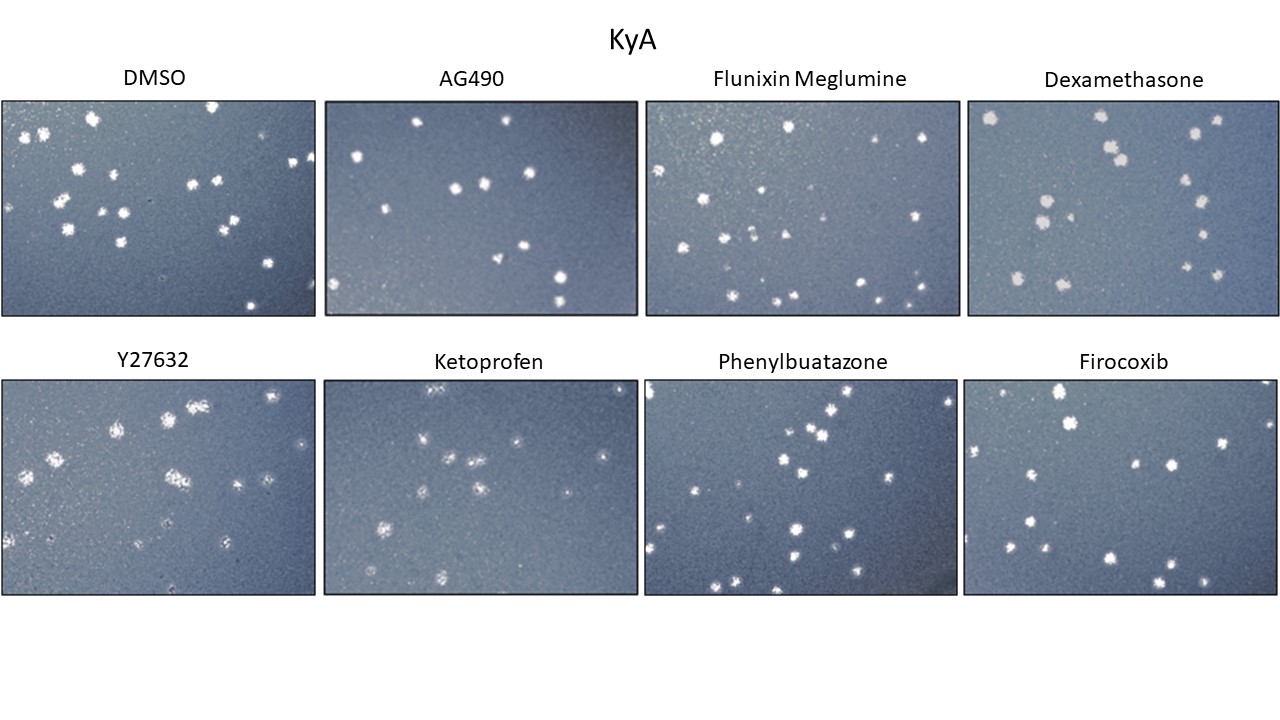

Supplement: Supplementary file 1 [file Image_1.JPEG]

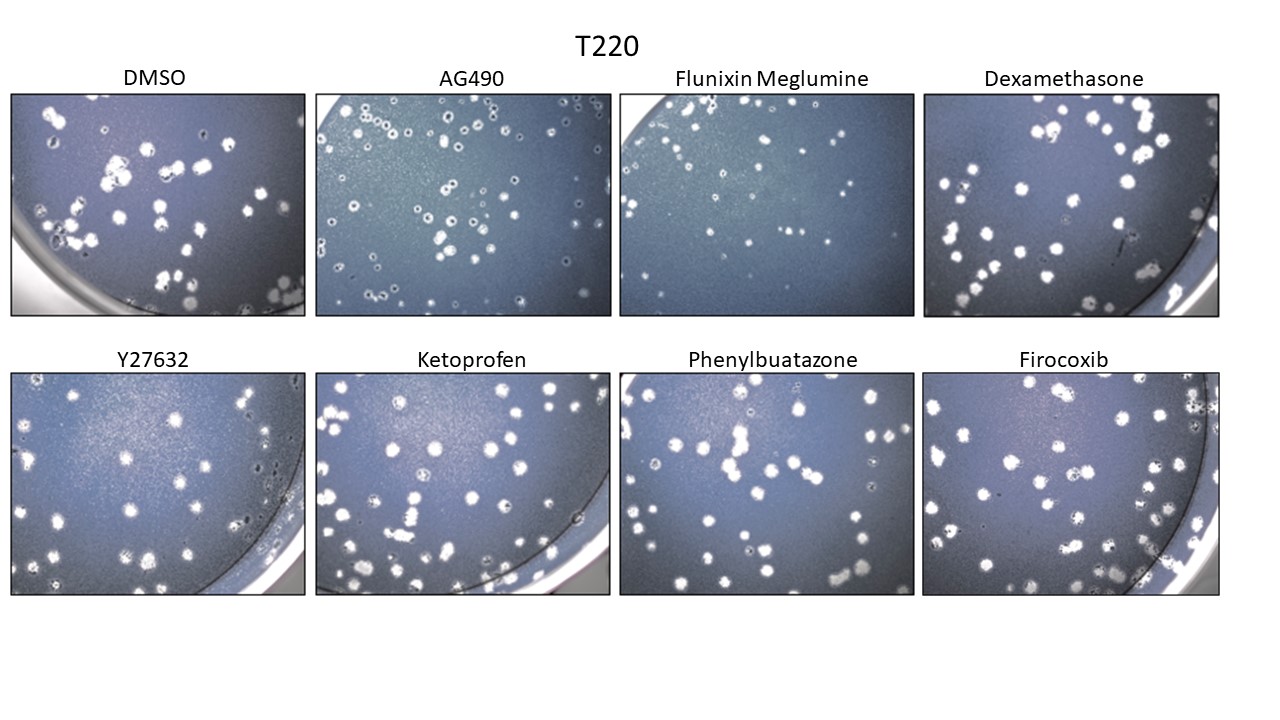

Supplement: Supplementary file 2 [file Image_2.JPEG]

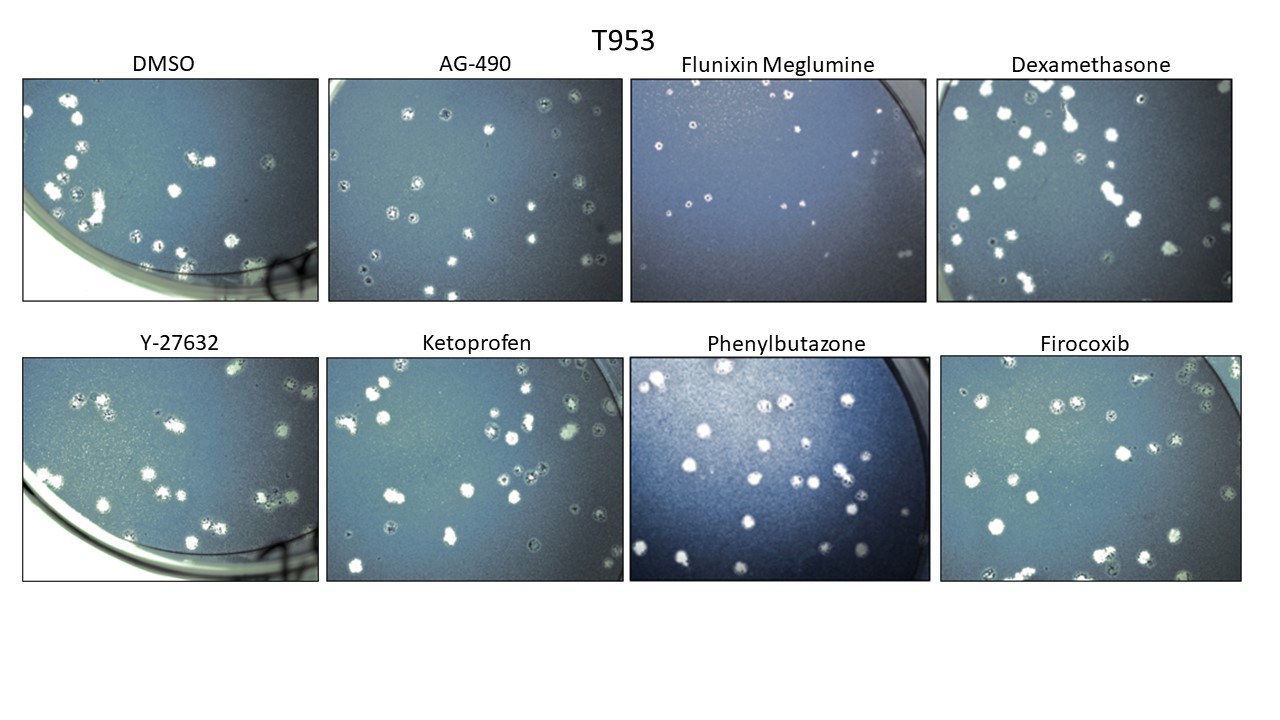

Supplement: Supplementary file 3 [file Image_3.JPEG]

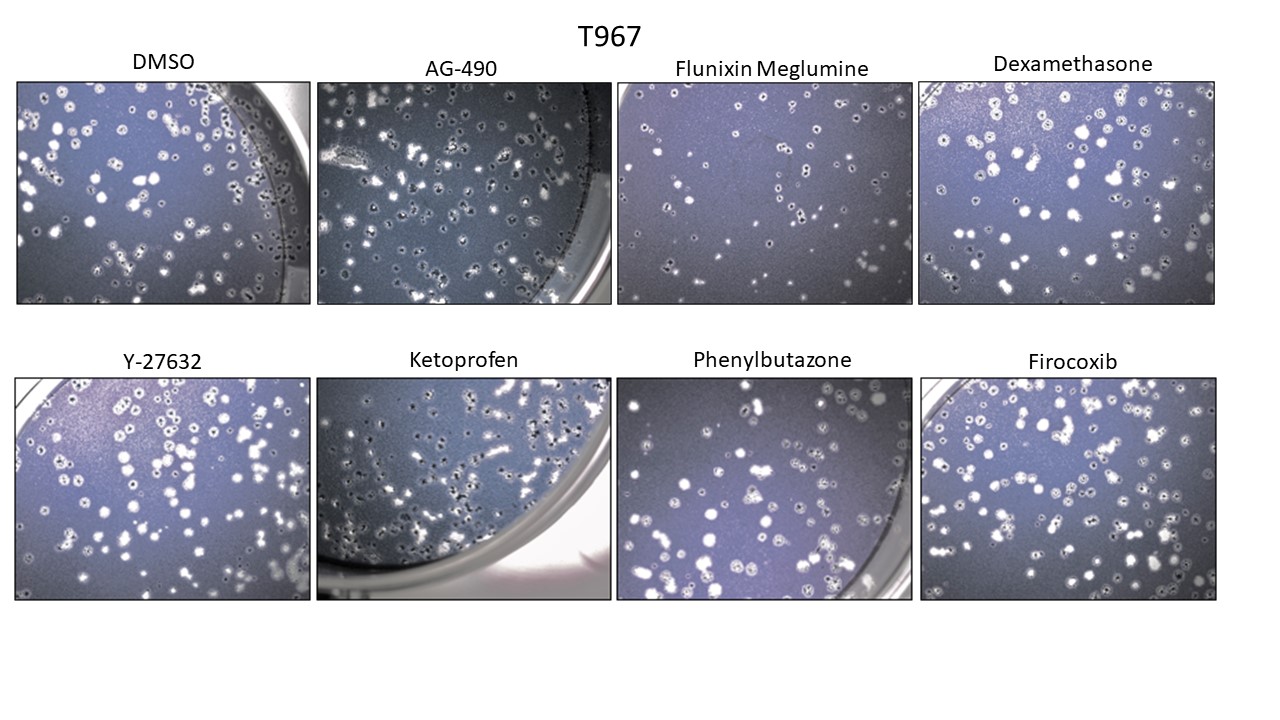

Supplement: Supplementary file 4 [file Image_4.JPEG]
